# Larval anatomy of *Dendropsophus decipiens* (A. Lutz 1925) (Anura: Hylidae: Dendropsophini) with considerations to larvae of this genus

**DOI:** 10.1371/journal.pone.0219716

**Published:** 2019-07-11

**Authors:** Pedro H. S. Dias, Katyuscia Araujo-Vieira, Ana Maria P. T. de Carvalho-e-Silva, Victor G. D. Orrico

**Affiliations:** 1 Departamento de Zoologia, Instituto de Biociências, Universidade de São Paulo, São Paulo, São Paulo, Brazil; 2 División Herpetología, Museo Argentino de Ciencias Naturales “Bernardino Rivadavia” – Conicet, Buenos Aires, Argentina; 3 Laboratório de Biossistemática de Anfíbios, Departamento de Zoologia, Universidade Federal do Estado do Rio de Janeiro, Rio de Janeiro, Rio de Janeiro, Brazil; 4 Departamento de Ciências Biológicas, Universidade Estadual de Santa Cruz, Salobrinho, Ilhéus, Bahia, Brazil; National Scientific and Technical Research Council (CONICET), ARGENTINA

## Abstract

The *Dendropsophus decipiens* clade comprises four species: *D*. *berthalutzae*, *D*. *decipiens*, *D*. *haddadi*, and *D*. *oliveirai*. Tadpoles of these species were described, but data on their internal morphology are lacking. We provide the first description of the buccopharyngeal anatomy, chondrocranial morphology, and cranial, hyoid and hyobranchial musculature of the tadpole of *D*. *decipiens*. Larvae of *D*. *decipiens* are characterized by the absence of lingual papillae, presence of fan-like papilla on the buccal floor, presence of a single-element suprarostral cartilage, presence of a small triangular process at the basis of the processus muscularis, m. levator mandibulae lateralis inserted on the nasal sac, and m. subarcualis rectus II-IV with a single, continuous slip. Tadpoles are likely macrophagous, although not as specialized as those of other species of the genus, suggesting some degree of diversification on the feeding habits within *Dendropsophus*.

## Introduction

Dendropsophini is a well-supported tribe of hylid treefrogs composed of the genera *Dendropsophus* and *Xenohyla* [1,2]. The relationships of Dendropsophini with other hylid tribes remain uncertain; it is poorly supported as sister taxon of Sphaenorhynchini [3] or as sister taxon of Pseudini [1,4–8]. *Dendropsophus* is a speciose clade of Neotropical treefrogs comprising 105 species distributed from Southern Mexico to Central-eastern Argentina [9], and the relationships between its species remain poorly known [1,3–8]. Three putative synapomorphies were suggested for the genus: diploid chromosome number of 30, the extreme reduction of quadratojugal, and labial tooth row formula 1/2 in tadpoles [3,10]. Nine species groups are recognized in *Dendropsophus*: the *D*. *columbianus*, *D*. *garagoensis*, *D*. *labialis*, *D*. *leucophyllatus*, *D*. *marmoratus*, *D*. *microcephalus*, *D*. *minimus*, *D*. *minutus*, and *D*. *parviceps* groups [3], whose composition have been slightly modified in the last few years (e.g. [11–13]).

The *Dendropsophus microcephalus* species group comprises more than 30 species, of which 13 are included in the two tentatively recognized clades: the *D*. *decipiens* and *D*. *rubicundulus* clades [3,11,13]. Two known morphological synapomorphies for this group are the absence of labial tooth rows and marginal papillae on the oral disc of larvae (with a reversal in the *D*. *decipiens* clade; [3,14]). The *D*. *microcephalus* group is the latest diverging taxon of *Dendropsophus*, and the relationship with other groups, as well as between its species remains controversial (e.g. [1,3,8]). The *D*. *decipiens* clade comprises four species: *D*. *berthalutzae*, *D*. *decipiens*, *D*. *haddadi*, and *D*. *oliveirai*. *Dendropsophus berthalutzae* is the only species of this clade included in molecular phylogenetic analyses (e.g. [1,3,15]), and therefore, the monophyly of the *D*. *decipiens* clade lacks a rigorous test. Putative synapomorphies for this clade are the presence of a posterior row of marginal papillae on the oral disc and oviposition on leaves overhanging water [3,16,17].

The external larval morphology in the *Dendropsophus decipiens* clade has been described for *D*. *berthalutzae* [18], *D*. *decipiens* [16], *D*. *haddadi* [19,20], and *D*. *oliverai* [16], but no aspect of their internal anatomy has been analyzed so far. We describe the buccopharyngeal anatomy, chondrocranial morphology, and cranial, hyoid and hyobranchial musculature of the tadpole of *D*. *decipiens*. We also provide comments on the larval internal morphology of *Dendropsophus* based on our observations complemented with data from the literature.

## Materials and methods

Tadpoles were collected (ICMBio/RAN permit # 13256–1) in a dam of Rio Borboleta (22°59'19"S, 44°06'13"W) at Reserva Rio das Pedras, Mangaratiba, Rio de Janeiro, Brazil. Specimens were euthanized by topical application of 20% benzocaine anesthetic mixed with water, preserved in 5% formaldehyde and deposited in the Coleção de Anfíbios do Laboratório de Biossistemática de Anfíbios da Universidade Federal do Estado do Rio de Janeiro (Lot UNIRIO 3635). Some tadpoles were raised in the laboratory to corroborate species identification. Developmental stages were determined according to Gosner [21].

Two individuals (stages 34 and 35) were dissected according to Wassersug [22] to expose the buccopharyngeal cavity. One individual (stage 34) was submitted to the protocol of Alcalde and Blotto [23] for scanning electron microscopy (SEM). Descriptive terminology follows Wassersug [22,24]. For observations of the chondrocranium and cranial muscles, six individuals (stages 30–36) were treated following the protocol of Dingerkus and Uhler [25] for clearing and staining; we interrupted the procedure just after the staining with alcian blue for two individuals (stage 36) which were then dissected manually for the study of larval muscles. After observations and illustrations, we finished the clearing protocol. Terminology for the chondrocranium and muscles follows Haas [26–28]. The character-states discussed through the text were optimized on the phylogenetic hypothesis proposed by Duellman et al. [1] with the software TNT v1.5 [29].

## Results

### External morphology

The tadpole of *Dendropsophus decipiens* (stage 37) was described by Pugliese et al. [16]. It is characterized by having a triangular body, high tail fins with brown stripes, and a reduced oral disc with blunt marginal papillae and absence of labial teeth.

### Buccopharyngeal anatomy

**Fig 1:**
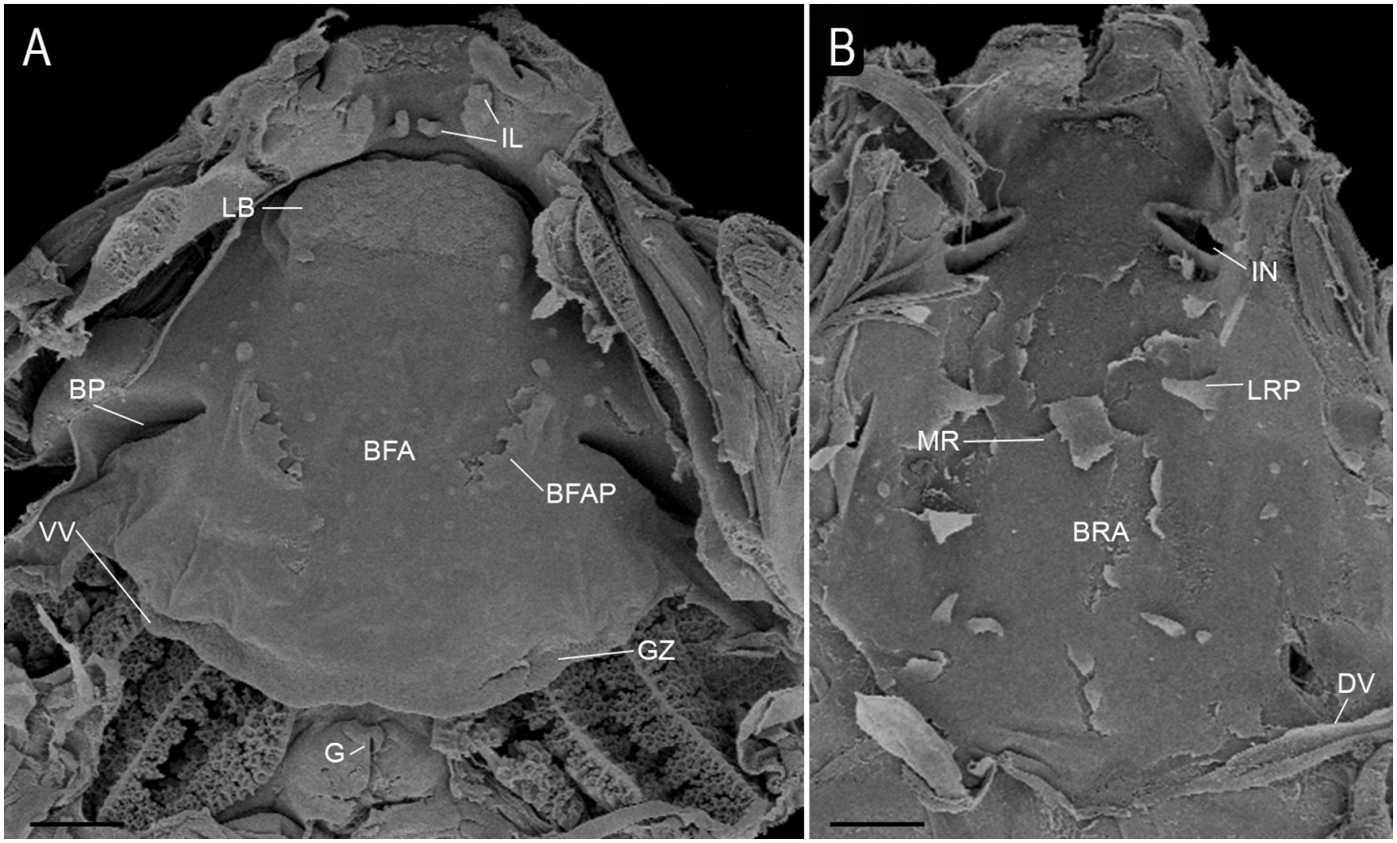
Buccopharyngeal anatomy of *Dendropsophus decipiens* (stage 34). (A) Buccal floor and (B) buccal roof. Abbreviations: BFA, buccal floor arena; BFAP, buccal floor arena papillae; BP, buccal pocket; BRA, buccal roof arena; DV, dorsal velum; G, glottis; GZ, glandular zone; IL, infralabial papillae; IN, internal nares; LB, lingual bud; LRP, lateral ridge papillae; MR, median ridge; VV, ventral velum. Scale bars = 400 μm.

Buccal floor triangular with two pairs of infralabial papillae; medial pair conical; lateral pair flap-like (Fig 1A). Lingual bud elliptical, lacking lingual papillae. Buccal floor arena U-shaped, laterally delimited by single fan-shaped papilla, with few pustulations. Prepocked pustulations present. Buccal pocket deep, oriented transversely. Glandular zone well-developed, with evident secretory pits, and well-marked spicular support. Ventral velum arch-shaped, lacking marginal projections; medial notch discreet. Branchial basket triangular, with three well-developed filter cavities. Filter plates unconnected, bearing many filter rows. Glottis exposed.

Buccal roof triangular (Fig 1B). Prenarial arena half-circle shaped, with few pustulations. Internal nares elliptical, oriented transversely; anterior border with four to five conical pustulations; posterior margin lacking valve. Postnarial papillae absent. Median ridge triangular, low. Lateral ridge papillae conical. Buccal roof arena poorly defined, delimited posterolaterally by two conical papillae. Glandular zone undistinguished. Dorsal velum arch-shaped, smooth, medially interrupted.

### Chondrocranium morphology

**Fig 2:**
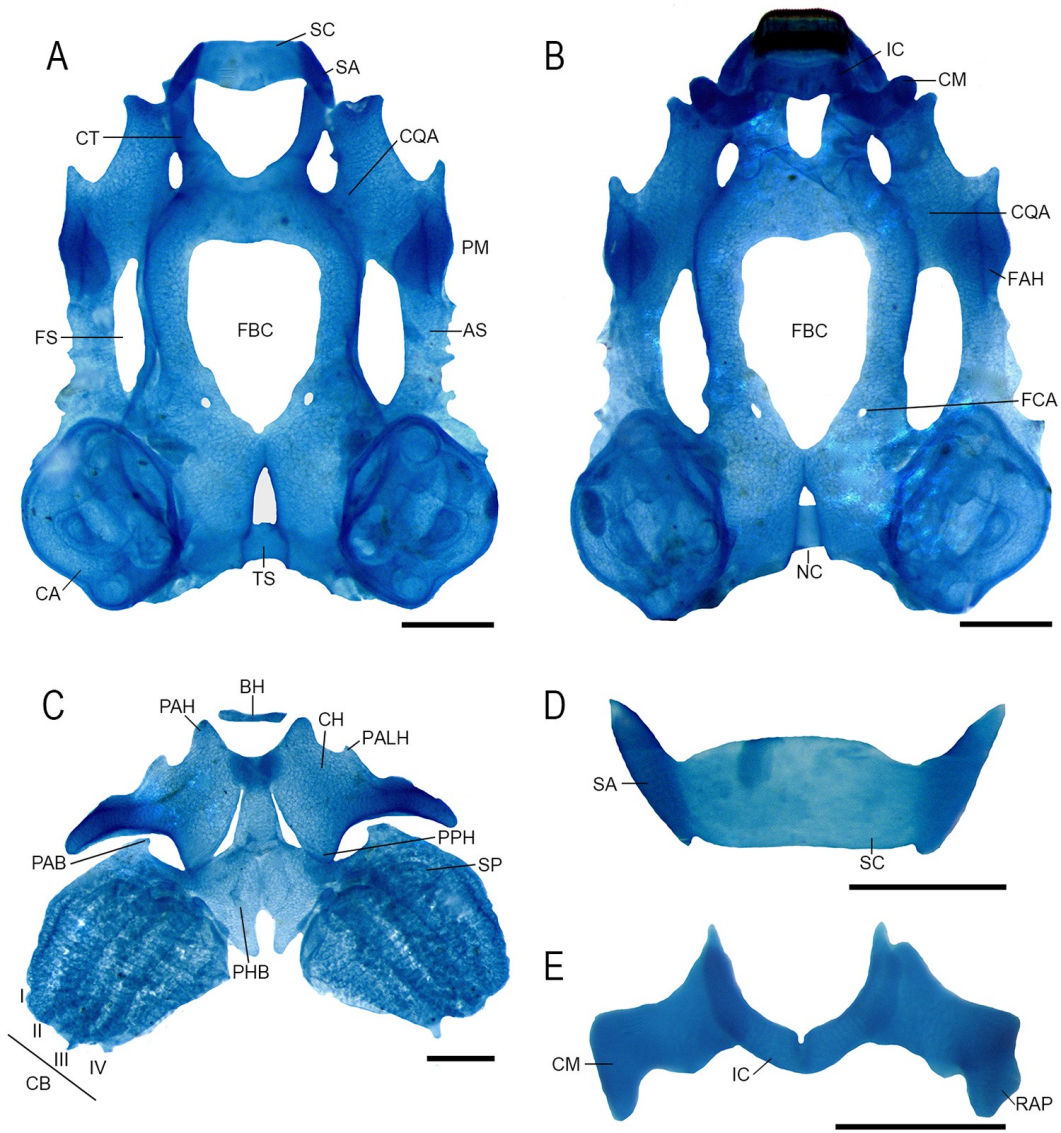
Chondrocranium morphology of *Dendropsophus decipiens* (stage 36). (A) Dorsal and (B) ventral views, (C) hyobranchial, (D) suprarostral cartilage, and (E) infrarostral and Cartilago Meckeli. Abbreviations: AS, arcus subocularis; BH, basihyal; CA, capsula auditivae; CB I-IV, ceratobranchials I-IV; CH, ceratohyal; CM, Cartilago Meckeli; CQA, commissura quadratocranialis anterior; CT, cornua trabeculae; FAH, facies articularis; FBC, fenestra basicranialis; FCA, foramen caroticum primarium; FS, fenestra subocularis; IC, infrarostral cartilage; NC, notochord; PAB, processus anterior branchiale; PAH, processus anterior hyalis; PALH, processus anterolateralis hyalis; PHB, planum hypobranchiale; PM, processus muscularis; PPH, processus posterior hyalis; RAP, retroarticular processus; SA, suprarostral alae; SC, suprarostral corpus; SP, spicules; TS, tectum synoticum. Scale bars = 0.5 mm.

Suprarostral cartilage single (Fig 2D); suprarostral alae and suprarostral corpus completely fused. Suprarostral alae triangular, bearing two processes: processus posterior dorsalis and anterior dorsalis. Cornu trabeculae short, thin, uniform along their extension, and parallel to each other; articulates with suprarostral (Fig 2A and 2B). Planum ethmoidale developed and distinct. Foramen nasalis present, elliptical. Fenestra basicranialis not occluded; planum intertrabeculare not formed in medial region; pierced by the foramen caroticum primarium.

Cartilago orbitalis tall, reaching the capsula auditivae posteriorly, forming the dorsal margin of the foramen prooticum; foramina oculomotorium, opticum, and prooticum pierce the cartilago orbitalis. Frontoparietal fontanelle large, rectangular and open (Fig 2A and 2B); laterally bordered by taenia tecti marginales, anteriorly by the planum ethmoidale, and posteriorly by the tectum synoticum. Capsula auditivaes robust, rhomboid-shaped, representing c.a. 28% of chondrocranium length. Anterolateral process of crista parotica absent. Jugulare and inferior perilymphaticum foramina present, visible in ventral view.

Palatoquadrate thin, long, slightly curved (Fig 2A and 2B); articular process articulate with cartilago Meckeli. Processes quadratoethmoidalis and pseudopterygoideus absent. Processus muscularis triangular, lower than cartilago orbitalis; triangular process present at the anterior margin of the process muscularis, triangular, anteriorly directed. Commissura quadratoorbitalis absent. Processus antorbitalis reduced. Subocular bar with small, triangular, lateral expansions. Processus ascendens attached to cranial floor with an angle of approximately 90°. Hyoquadrate process evident on ventral surface of processus muscularis. Cartilago Meckeli sigmoid-shaped, orientated almost perpendicular to chondrocranium axis (Fig 2E). Infrarostral cartilages rectangular, curved, joined medially by connective tissue.

Ceratohyals long, flat, and subtriangular (Fig 2C); their anterior margin bearing two triangular, well-developed processes: processus anterior hyalis and processus anterolateralis hyalis. Posterior processes triangular, tall. Ceratohyals are joined by a pars reuniens, which is chondrified. Basibranchial rectangular, bearing a rounded processus urobranchialis. Basihyal present, slim, cylindrical. Planum hypobranchiale long, triangular, contacting each other along their anterior half. Branchial basket has four curved ceratobranchials with numerous lateral projections. Ceratobranchial I continuous with the planum hypobranchiale; dorsally, it bears a triangular processus anterior branchialis. Ceratobranchials II and III also fuse to the planum hypobranchiale plate and bear round branchial processes. Ceratobranchial IV is shorter, wider, and fused to the planum hypobranchiale. Four long, curved spicules develop dorsally; the third and fourth spicules are connected to the planum hypobranchiale by a thin cartilaginous bridge. Ceratobranchials are distally joined by terminal commissures.

### Cranial, hyoid and hyobranchial musculature

**Fig 3:**
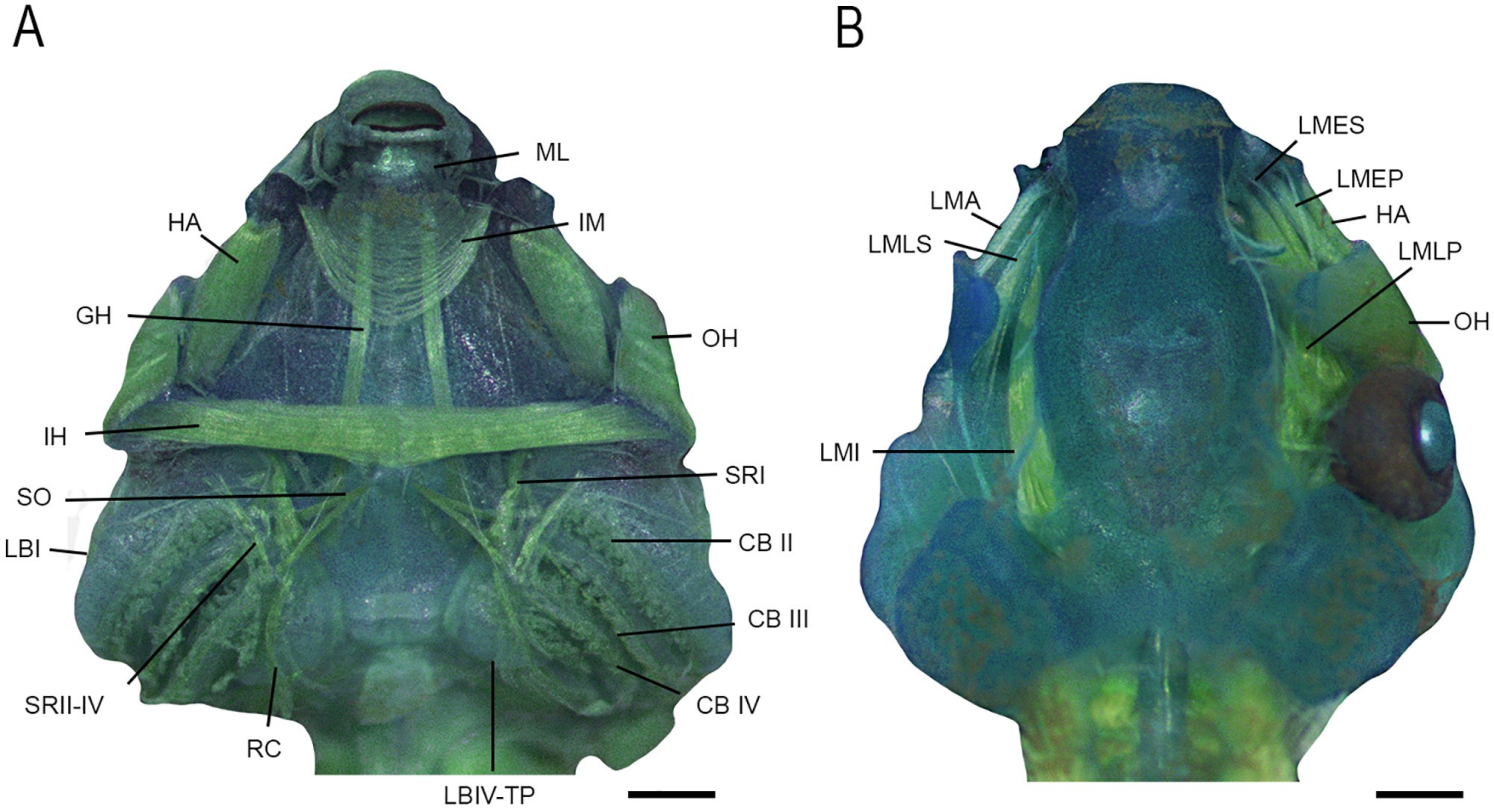
Cranial, hyoid and hyobranchial muscles of *Dendropsophus decipiens* (stage 36). (A) Ventral and (B) dorsal views. Abbreviations: CB (II-IV), constrictor branchialis; GH, genyohioideus; HA, hyoangularis; IH, interhyoideus; IM, intermandibularis; LBI, levator arcuum branchialium I; LBIV-TP, levator arcuum branchialium IV + tympanopharyngeus; LMA, levator mandibulae articularis; LMEP, levator mandibulae externus profundus; LMES, levator mandibulae externus superficialis; LMI, levator mandibulae internus; LMLP, levator mandibulae longus profundus; LMLS, levator mandibulae longus superficialis; ML, mandibulolabialis; OH, orbitohyoideus; RC, rectus cervicis; SO, subarcualis obliquus; SRI, subarcualis rectus I; SRII-IV, subarcualis II-IV. Scale bars = 0.5 mm.

**Fig 4:**
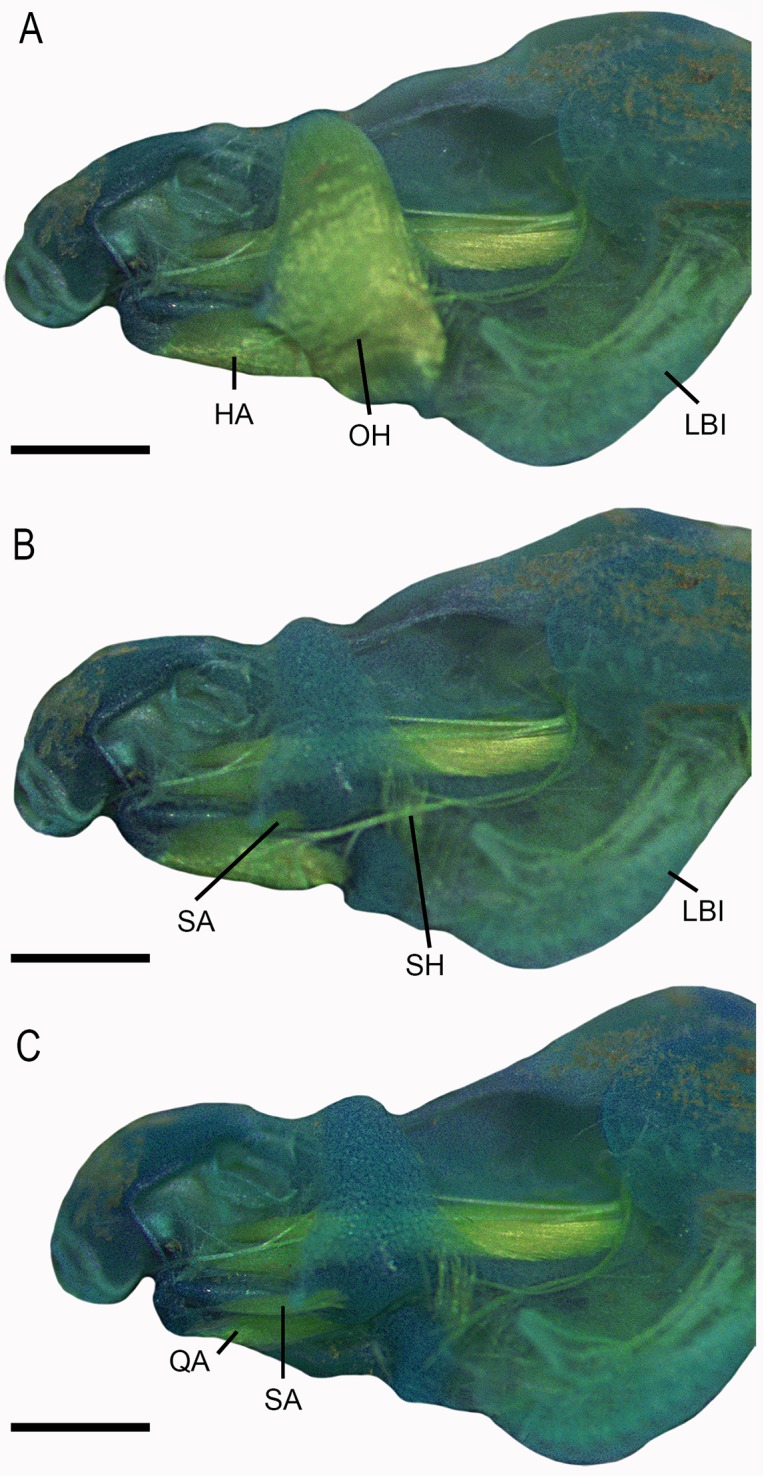
Lateral view of cranial, hyoid and hyobranchial muscles of *Dendropsophus decipiens* (stage 36). Abbreviations: HA, hyoangularis; LBI, levator arcuum branchialium I; OH, orbitohyoideus; QA, quadratoangularis; SA, suspensorioangularis; SH, suspensoriohyoideus. Scale bars = 0.5 mm.

We found a total of 31 muscles in larvae of *Dendropsophus decipiens* (origins and insertions in Table 1; Figs 3 and 4). Interhyoideus posterior and diaphragmatopraecordialis absent. Subarcualis obliquus present in two slips, inserting on the processus branchialis II and III. Subarcualis rectus II-IV continuous, inserting on the ceratobranchial I. Mandibulolabialis with single slip, corresponding to mandibulolabialis inferior. Intermandibularis arch-shaped. Larval levator mandibulae externus with two slips, superficialis and profundus. Levator mandibulae lateralis inserting in the nasal sac. Ramus mandibularis (cranial nerve V_3_) runs dorsally to longus and externus groups.

**Table 1:** Cranial, hyoid and hyobranchial musculature of the tadpole of *Dendropsophus decipiens* (stage 36). Abbreviations: CB I, II, and III = Ceratobranchial I, II, and III. c.n. = cranial nerve. LMLP = m. levator mandibulae longus profundus. n. = nerve.

Muscle	Origin	Insertion	Comments
**Mandibular group, n. trigeminus (c.n. V) innervated**			
Levator mandibulae longus superficialis	External posterior margin arcus subocularis and processus ascendens	Dorsomedial cartilago Meckeli	Via long tendon
Levator mandibulae longus profundus	External margin (curvature) arcus subocularis	External margin of the suprarostral ala	Via a long tendon; tissue mass near the upper lip
Levator mandibulae externus superficialis	Inner processus muscularis (superior)	Processus posterior dorsalis of suprarostral ala	
Levator mandibulae externus profundus	Inner processus muscularis (medial)	External, anteroventral margin of suprarostral ala	Share a tendon with LMLP
Levator mandibulae articularis	Inner processus muscularis (inferior)	Dorsal cartilago Meckeli	
Levator mandibulae lateralis	Processus articularis quadrati	Nasal sac	
Submentalis (intermandibularis anterior)	-	-	
Intermandibularis	Median aponeurosis	Ventromedial cartilago Meckeli	
Mandibulolabialis	Ventromedial cartilago Meckeli	Lower lip	Single slip (inferior)
Levator mandibulae internus	Ventral processus ascendens and few fibers on the lateral curvature	Distal cartilago Meckeli	
**Hyoid group, n. facialis (c.n. VII)**			
Hyoangularis	Dorsal ceratohyal	Retroarticular process of cartilago Meckeli	
Quadratoangularis	Ventral palatoquadrate	Retroarticular process of cartilago Meckeli	
Suspensorioangularis	Descendent posterior margin of the processus muscularis	Retroarticular process of cartilago Meckeli	
Orbitohyoideus	Processus muscularis	Lateral edge of ceratohyal	
Suspensoriohyoideus	Posterior descending margin of processus muscularis	Lateral process of ceratohyal	
Interhyoideus	Median aponeurosis	Ventral ceratohyal	
**Branchial group, n. glossopharyngeus (c.n. IX) and vagus (c.n. X)**			
Levator arcus branchialium I	Arcus subocularis	Ceratobranchial I	
Levator arcus branchialium II	Lateral otic capsule	Ceratobranchial II	
Levator arcus branchialium III	Lateral otic capsule	Ceratobranchial III	
Levator arcus branchialium IV	Otic capsule	Ceratobranchial IV	
Tympanopharyngeus	Otic capsule	Ceratobranchial IV and pericardium	
Dilator laryngis	Otic capsule	Arytenoid cartilage	
Constrictor branchialis I	-	-	
Constrictor branchialis II	Processus branchialis II	Commissura terminalis I	by the inner margin of CB I
Constrictor branchialis III	Processus branchialis II	Commissura terminalis II	by the inner margin of CB II
Constrictor branchialis IV	Processus branchialis II	Commissura terminalis II	Along the CB III
Subarcualis rectus I	Posterior lateral base of ceratohyal	Processus branchialis III, dorsal CB II and CB I	
Subarcualis rectus II-IV	Ceratobranchial IV	Ceratobranchial I	Crossing the CB III
Subarcualis obliquus II	Processus urobranchialis	Processus branchialis II and III	Two slips
Diaphragmatobranchialis	Peritoneum (diaphragm)	Distal Ceratobranchial III	
**Spinal group, spinal nerve innervation**			
Genyohioideus	Planum hypobranchiale	Infrarostral cartilage	Originating between CB II and III
Rectus abdominis	Peritoneum (diaphragm)	Abdominal wall	Five open myotomes
Rectus cervices	Peritoneum (diaphragm)	Processus branchialis III	Single slip

## Discussion

### Larval internal morphology

*Dendropsophus* has been repeatedly found as sister taxon of *Xenohyla* (always well supported) in molecular phylogenetic analyses for Hylidae (e.g. [1,3,6,8]). However, the phylogenetic relationships of the tribe Dendropsophini (*Dendropsophus* + *Xenohyla*; sensu [2]) within Hylidae remain controversial. It is recovered as sister taxon of Sphaenorhynchini, closely related to Pseudini and Scinaxini [3] or alternatively, as sister taxon of Pseudini, distantly related to Scinaxini and Sphaenorhynchini (e.g. [1,8]).

Descriptions of the internal larval anatomy are scarce for *Dendropsophus*. The buccopharyngeal anatomy is known for *D*. *ebraccatus*, *D*. *garagoensis*, *D*. *minutus*, *D*. *microcephalus*, *D*. *nanus*, *D*. *padreluna*, *D*. *phlebodes*, *D*. *sarayacuensis*, *D*. *stingi*, and *D*. *virolinensis* [24,30–32]. Similarly, descriptions of cranial muscles and the chondrocranium morphology are only available for *D*. *ebraccatus*, *D*. *microcephalus*, and *D*. *nanus* [28,32–34]. *Dendropsophus ebraccatus* was included in the phylogenetic analyses of Haas [28]. Aside from those studies, there is some scattered information available as the relative size of the buccal depressors muscles of *D*. *microcephalus* and *D*. *phlebodes* [35], buccal pumping anatomy and secretory ridges of *D*. *microcephalus* [36,37], hyobranchial morphology of *D*. *nanus* [38], and some character-states of chondrocranium for *D*. *microcephalus* [39].

The controversial phylogenetic position of the tribe Dendropsophini (e.g. [1,3]) within Hylidae, added to the poor knowledge about on internal larval anatomy in *Dendropsophus* prevents us to assess the polarity of most character-states and, therefore, to enhance broad discussions about larval character evolution in *Dendropsophus*; however, there are some interesting exceptions that deserve comments.

Several species of *Dendropsophus* have a reduced number of elements in the buccopharyngeal cavity [24,30–32]. For example, the median ridge is missing in *D*. *ebraccatus* and *D*. *nanus* [24,32], and a lower number of papillae in both buccal floor and roof and the absence of lingual papillae were also reported for larvae of *Dendropsophus* (e.g. [31,32]). Faivovich et al. [3] suggested the absence of lingual papillae as a putative synapomorphy for the clade composed of *Dendropsophus*, *Lysapsus*, *Pseudis*, *Scarthyla*, *Scinax*, and *Sphaenorhynchus* (the former Dendropsophini tribe sensu [3]), with reversion in *Lysapsus* and *Pseudis*. Our observations showed that lingual papillae are also present at least in some species of *Sphaenorhynchus* (*S*. *dorisae*, *S*. *lacteus*, *S*. *prasinus*; P.H.S. Dias and K. Araujo-Vieira personal observations). This character-state is unknown for *Xenohyla*. The absence of lingual papillae optimizes ambiguously in the phylogenetic hypothesis of Duellman et al. [1] due to the unknown character-state of *Xenohyla*; it is a synapomorphy of *Dendropsophus* or of a more inclusive clade, the tribe Dendropsophini (*Dendropsophus* + *Xenohyla* sensu [2]).

*Dendropsophus decipiens* (*D*. *microcephalus* group) has a fan-like papilla on the buccal floor, adjacent to the buccal pockets. This character was first described by Kaplan and Ruíz-Carranza [31] for *D*. *garagoensis*, *D*. *padreluna*, *D*. *virolinensis* (*D*. *garagoensis* group), and *D*. *stingi* (*D*. *parviceps* group) and illustrated for *D*. *minutus* (*D*. *minutus* group; [30]). Fan-like papillae are absent in larvae of *D*. *ebraccatus*, *D*. *microcephalus*, *D*. *nanus*, *D*. *phlebodes*, and *D*. *sarayacuensis* [24,28,32]. The presence of a fan-like papilla on the buccal floor in members of the *D*. *garagoensis*, *D*. *microcephalus*, *D*. *minutus*, and *D*. *parviceps* groups suggest that it could be a putative synapomorphy of the genus *Dendropsophus*; however, the taxonomic distribution of this character-state within *Dendropsophus* remains poorly known.

A putative synapomorphy of the *Dendropsophus garagoensis* group is the presence of two pairs of infralabial papillae, which were reported for *D*. *garagoensis* and *D*. *padreluna* [31]—with independent evolution in *D*. *decipiens* [this work]. Other species of *Dendropsophus* that have been studied present a single pair of infralabial papillae (e.g. *D*. *minutus*, *D*. *nanus*, *D*. *phlebodes*; [24,30,32]).

Larvae of *Dendropsophus decipiens* differ from those of its related species *D*. *microcephalus* and *D*. *nanus* (characters in parenthesis) in having generalized processus muscularis (broad processus muscularis), marginal projections on arcus subocularis of palatoquadrate (smooth margin), thin ceratohyals (stout and thick ceratohyals), regular branchial basket (reduced branchial basked), and presence of spicules on the ceratobranchialia (absence of spicules) [32–34]. However, *D*. *decipiens* share with those species short and narrow cornu trabeculae, the presence of a small triangular process at the basis of the processus muscularis, and a single-element suprarostral cartilage. The latter could be a synapomorphy of *Dendropsophus* (with one reversion to a separated element suprarostral cartilage in *D*. *ebraccatus* within the *D*. *leucophyllatus* group; [28]) or of the *D*. *microcephalus* group. In larvae of *Lysapsus*, *Pseudis*, and some *Scinax*, the suprarostral corpora are fused distally, but with a medial indentation that allow the identification of both elements; several other hylids have completely separated suprarostral cartilage (e.g. [32,33,40–43]). This character-state is still unknown for *Scarthyla*, *Sphaenorhynchus*, and *Xenohyla*.

It is worth to note that a single-element suprarostral cartilage is not very common in anuran larvae. Besides some *Dendropsophus*, such morphological condition have been reported in some microhylids [32], *Occidozyga baluensis* (Dicroglossidae) [44], and *Ceratophrys* and *Lepidobatrachus* (Ceratophryidae) [32,45–47]. It is interesting to point that, regarding Ceratophryidae, *Chacophrys pierotti* possesses the suprarostral alae and corpora completely fused (with a small fenestra), but both corpora have a medial indentation [48]. Lavilla and Fabrezi [49] suggested that the complete fused suprarostral could be a synapomorphy of *Ceratophrys* + *Lepidobatrachus*. Given the phylogenetic relationships within Ceratophryidae [50]—*Chacophrys* and *Lepidobatrachus* are sister taxa, and both sister to *Ceratophrys*—this is not possible. However, the complete fusion between the alae and corpora could be a synapomorphy for the family. While microhylids are filter-feeders, *Ceratophrys*, *Lepidobatrachus*, and *Occidozyga* are macrophagous larvae. Further information are still needed to understand the relationship between macrophagy and the fusion of the elements of the suprarostral cartilage.

*Dendropsophus decipiens* shares with *D*. *ebraccatus* the m. subarcualis rectus II-IV with a single, continuous slip; whereas *D*. *microcephalus* and *D*. *nanus* have this muscle discontinued at the processus branchialis II [28,32,41]. Larvae of *Scinax* and distantly related hylids (e.g. *Agalychnis*, *Boana*, *Osteocephalus*, *Trachycephalus*; [28,32,42]) have a single subarcualis rectus II-IV, while larvae of *Lysapsus* and *Pseudis* have a discontinued subarcualis rectus II-IV [28,32,34]. The taxonomic distribution of these character-states on the phylogenetic hypothesis of Duellman et al. [1] suggests that *D*. *decipiens* and *D*. *ebraccatus* have the plesiomorphic condition (single subarcualis rectus II-IV), being the m. subarcualis rectus II-IV discontinued at the processus branchialis II a putative synapomorphy of *Lysapsus* + *Pseudis*, with instances of homoplasy in *D*. *microcephalus* and *D*. *nanus*.

Larvae of *Dendropsophus*, *Lysapsus*, and *Pseudis* that have been studied share the m. levator mandibulae lateralis inserted on the nasal sac [32,41] [this work]—although Haas [28] mentioned that it inserted on the processus posterior dorsalis of the suprarostral or in the rostral tissue in *D*. *ebraccatus* [28: ch. 57.0]. In larvae of *Scinax*, the m. levator mandibulae lateralis inserts on the pars alaris of the suprarostral cartilage [32,34,42]. The taxonomic distribution of these character-states on the phylogenetic hypothesis of Duellman et al. [1] suggests that the m. levator mandibulae lateralis inserted on the nasal sac could be a synapomorphy of the Dendropsophini + Pseudini clade (sensu [2]) or arose twice independently in *Dendropsophus* and the *Lysapsus* + *Pseudis* clade. Other character-state, the absence of the superior slip of the m. mandibulolabialis also optimize ambiguously as a synapomorphy of the Dendropsophini + Pseudini clade or of *Dendropsophus* and the *Lysapsus* + *Pseudis* clade (with some instances of reversion within *Pseudis*; [28,41]). Larvae of *Scinax* have the superior slip of the m. mandibulolabialis [42]. The optimizations of both character-states mentioned above are dependent on the conditions, still unknown, present in larvae of *Scarthyla*, *Sphaenorhynchus*, and *Xenohyla*.

### Feeding habits

Tadpoles can be distinguished regarding their deeding habits accordingly with the size of their prey (macrophagous or microphagous) and the nature of the feeding items (herbivore, carnivore, or omnivore). Larvae of *Dendropsophus* have been described as macrophagous feeders (e.g. [51]), or macrophagous herbivores according to Wassersug [24]. However, other reports suggest that tadpoles of *Dendropsophus* could be omnivorous. For example, Vera Candioti [32] described the presence of entire oligochaetes in the digestive system of *D*. *nanus*. Ruas et al. [52] observed tadpoles of *D*. *novaisi* chasing and preying on tadpoles of *Rhinella crucifer*. Also, larvae of *D*. *minutus* were observed attacking and preying on living tadpoles of *Physalaemus* sp.—although it was considered a facultative behavior, given that *D*. *minutus* feeds commercial fish food and plant residues in captivity [53].

Most of the macrophagous tadpoles (both herbivorous or carnivores) have terminal mouth, reduced branchial basket, well-developed orbitohyoideus, reduced labial tooth rows, reduced or lost elements of the buccopharynx, massive jaw sheaths, fused elements of suprarostral cartilages, and short intestines [32,35–36,44–47,54] [P.H.S. Dias personal observation]. Almost all these character-states are present, with different combinations, in larvae of *Dendropsophus*. Wassersug [24] pointed out several buccopharyngeal characters associated with macrophagy in larvae of *Dendropsophus*, and also reported that *D*. *phlebodes* “presented the most extreme reduction in structures associated with fine suspended matters”.

We observed that larvae of *Dendropsophus decipiens* have some character associated with a macrophagy as a terminal mouth lacking labial teeth, reduction in the elements of the buccopharyngeal cavity, such as papillation and secretory tissue, and single-element suprarostral cartilage. Nevertheless, tadpoles of *D*. *decipiens* differ from those of *D*. *microcephalus* and *D*. *nanus*, which have marked reduction of branchial baskets (well-formed and bearing spicules in *D*. *decipiens*) and massive orbitohyoideus [32] that is correlated with powerful suction capacity [35]. This combination of characters suggests that larvae of *D*. *decipiens* feed on large elements and have reduced filter capacity, although they are not as specialized for predation (carnivory/omnivory) as the tadpoles of *D*. *microcephalus* and *D*. *nanus*. Morphological and behavioral observations for more species, as well as the usage of techniques such as stable isotope (see [55]) are necessary for a fully appreciation of the evolution of feeding habits in *Dendropsophus*.

Our results suggest that larval internal morphology could provide interesting insights into the evolution and diversification of *Dendropsophus*. However, additional studies on other species of *Dendropsophus* and its related taxa (e.g. *Scarthyla*, *Sphaenorhynchus*, and *Xenohyla*) are required to corroborate the different putative synapomorphies mentioned in this work. Further, the feeding habits of tadpoles of the genus are, putatively, more diverse than previously observed, with variable degrees of specialization for a macrophagous diet.
